# Knowledge, attitude, and practice toward sleep hygiene and cardiovascular health: a cross-sectional survey among healthcare workers

**DOI:** 10.3389/fpubh.2024.1415849

**Published:** 2024-10-17

**Authors:** Weixin Sun, Renyou Pan, Xiaolong Song, Tingting Gu, Qimeng Ni, Yuexing Gu

**Affiliations:** ^1^Department of Cardiology, Yancheng TCM Hospital Affiliated to Nanjing University of Chinese Medicine, Yancheng, Jiangsu, China; ^2^Department of Cardiology, Yancheng TCM Hospital, Yancheng, Jiangsu, China

**Keywords:** knowledge, attitude, practice, healthcare professional, chronic insomnia, sleep health awareness, sleep patterns, cross-sectional study

## Abstract

**Background:**

Healthcare workers grapple with distinct challenges, inherent to their profession, making them susceptible to irregular sleep patterns and insufficient sleep, which may further impact their cardiovascular health. This study aimed to investigate the knowledge, attitude and practice (KAP) of healthcare workers concerning sleep hygiene and cardiovascular health.

**Methods:**

A cross-sectional survey was conducted at Yancheng TCM Hospital Affiliated with Nanjing University of Chinese Medicine between July, 2023 and September, 2023. Demographic characteristics, KAP scores, sleep health awareness and habits, and sleep quality were assessed through the questionnaires.

**Results:**

A total of 423 valid questionnaires were included in the study. Among these, 215 (50.83%) were aged 35 or below, and 128 (30.26%) reported suffering from chronic insomnia. The mean knowledge, attitude, and practice scores were 43.23 ± 5.67 (possible range: 10–50), 24.53 ± 4.59 (possible range: 9–45), and 20.22 ± 4.46 (possible range: 6–30), respectively. Multivariate analyses indicated that attitude score (OR = 0.890, 95% CI: [0.807–0.981], *p* = 0.019), sleep health awareness & habits score (OR = 1.847, 95% CI: [1.284–2.656], *p* = 0.001), experienced chest tightness or heart palpitations (OR = 6.084, 95% CI: [2.172–17.042], *p* = 0.001), and the presence of chronic insomnia (OR = 5.017, 95% CI: [2.428–10.368], *p* < 0.001) were independently associated with sleep quality.

**Conclusion:**

Healthcare workers had adequate knowledge, negative attitude and moderate practice toward sleep hygiene and cardiovascular health. The findings highlight the need for targeted interventions to address the observed gaps in attitudes and practices, aiming to enhance overall awareness and promote healthier sleep habits among healthcare professionals.

## Introduction

Cardiovascular diseases pose a significant global health challenge, ranking as a leading cause of both morbidity and mortality (1, 2). The intricate relationship between sleep and cardiovascular health has emerged as a key focus in public health research. Inadequate sleep, identified as a pivotal factor, is not only associated with the development but also the exacerbation of cardiovascular diseases. A previous study have highlighted how sleep disturbances serve as precursors to a range of health problems, including cardiovascular diseases (3). For instance, research indicates that insufficient sleep substantially increases the risk of stroke. A notable study revealed that individuals sleeping fewer than 5 h per night face a significantly elevated risk, with evidence suggesting a threefold increase in stroke risk in cases of chronic sleep deprivation (4). Sleep, considered as a fundamental physiological process, plays a critical role in maintaining cardiovascular homeostasis (5). The regulation of sleep involves distinct components, including a sleep-promoting, homeostatic element and a circadian component (6). The homeostatic process involves the gradual accumulation of sleep pressure during waking hours, which dissipates during the sleep phase. This intricate interplay between sleep homeostasis and circadian rhythm extends beyond cardiovascular health, also exerting significant influence on mental health and pathology (7). The adverse effects of inadequate sleep extend to various cardiovascular parameters, such as blood pressure, endothelial function, and metabolic regulation (8). Furthermore, abnormal sleep duration or poor sleep quality resulting from sleep disorders can precipitate a spectrum of health issues, spanning cardiovascular, metabolic, neurological, and malignant diseases (9).

Effective sleep hygiene, which includes principles such as maintaining a regular sleep schedule, creating a conducive sleep environment, and avoiding sleep-disruptive behaviors like caffeine or screen exposure before bed, plays a vital role in promoting restorative sleep and reducing the risks associated with poor sleep. These principles are particularly important in mitigating the adverse cardiovascular outcomes linked to inadequate sleep. Research shows that adherence to these sleep hygiene practices can significantly improve sleep quality, which in turn contributes to better cardiovascular health outcomes (10). Therefore, public health initiatives and educational programs that focus on improving sleep hygiene are instrumental in reducing the incidence of cardiovascular diseases and enhancing cardiovascular health outcomes.

Healthcare workers grapple with distinct challenges inherent to their profession, making them susceptible to irregular sleep patterns and insufficient sleep, as evidenced by a recent study (11). The demanding nature of their occupation, characterized by stringent schedules, shift work, and exposure to occupational stressors, significantly heightens the risk of sleep disturbances. Previous clinical trial and cross-sectional study have revealed that over 40% of healthcare practitioners, including nurses and residents, report inadequate sleep (12, 13). Notably, healthcare professionals typically obtain only 4–7 h between night shifts, a practice associated with sleep deprivation, fatigue, and performance deficits (14). Knowledge, Attitude, and Practice (KAP) studies are crucial for exploring individuals’ understanding, perceptions, and behaviors on specific health-related topics, serving as valuable tools (15). Grounded in health literacy principles, these studies posit that knowledge positively influences attitudes, which, in turn, shape individual practices (16). Applying the KAP framework to investigate healthcare workers’ perspectives on sleep and cardiovascular health is paramount. Such exploration is vital for identifying targeted interventions and preventive strategies to address the unique sleep challenges faced by healthcare professionals.

Despite the recognized significance of sleep in cardiovascular health, there is a research gap in comprehensively understanding the KAP of healthcare workers concerning sleep hygiene and cardiovascular health. Previous studies have demonstrated that physical therapists recognize the impact of sleep on patient outcomes and acknowledge the necessity of assessing patients’ sleep conditions and receiving education on sleep hygiene (17). However, the awareness of healthcare professionals, including nurses, about the importance of sleep hygiene and cardiovascular health remains unclear. Although nurses are aware of the significance of healthy behaviors, this knowledge does not always translate into self-care practices (18). This discrepancy suggests a gap in the application of knowledge to personal health management among healthcare workers. Therefore, this study aims to investigate the KAP of healthcare workers concerning sleep and cardiovascular health.

## Materials and methods

### Study design and participants

This cross-sectional study was conducted at Yancheng TCM Hospital, affiliated with Nanjing University of Chinese Medicine, between July 2023 and September 2023, and involved healthcare workers as participants by convenience sampling. The inclusion criteria encompassed: (1) Healthcare professionals with certifications, including doctors, nurses, pharmacists, technicians, and administrative personnel, and (2) expressing willingness to participate in the study. The exclusion criteria encompassed: (1) Refuse to participate; (2) furnishing response with logical errors. This study was approved by the Ethic Committee of Yancheng TCM Hospital (Ethics Approval Number: ky230513), and all participants provided written informed consent.

### Questionnaire

The questionnaire was initially developed based on previous studies (19–22). Following the initial design, feedback from five experts, including 2 cardiovascular specialists, 2 psychology experts, and 1 biostatistics expert, was incorporated to refine the questionnaire, to ensure the content and face validity of the questionnaire. And a pilot study among 55 participants indicated an acceptable internal consistency (Cronbach’s *α* = 0.800). In the formal survey, the reliability statistics were further confirmed, with Cronbach’s α values of 0.862 for the overall questionnaire, and 0.905, 0.753, and 0.770 for the Knowledge, Attitudes, and Practices sections, respectively.

The finalized KAP questionnaire was in Chinese, and comprises 4 distinct dimensions: demographic characteristics, the knowledge dimension, the attitude dimension, and the practice dimension. Additionally, we utilized the Sleep Hygiene Awareness and Habit Scale (SHAPS) and the Pittsburgh Sleep Quality Index (PSQI), both of which have proven to be effective in previous studies among the Chinese population (23, 24). The SHAPS evaluates awareness regarding twelve factors that adversely affect sleep. It employs a Likert-type scale for responses, ranging from ‘1’ (indicating ‘very beneficial’) to ‘7’ (indicating ‘very disruptive’), with intermediate values representing varying degrees of benefit or disruption to sleep (2 = moderately beneficial, 3 = mildly beneficial, 4 = no effect, 5 = mildly disruptive, 6 = moderately disruptive). Higher scores on the SHAPS indicate greater awareness of behaviors and actions that negatively impact sleep quality (25, 26). Previous research has demonstrated that it exhibited strong reliability and validity (27, 28). In PSQI scoring, seven components should be considered. The minimum and maximum scores for each component are zero (no problem) and three (very serious problem), respectively. A higher score indicates poorer sleep quality. Specifically, a total score ≤ 5 points indicates good sleep quality, a total score of 6–10 points indicates average sleep quality, a total score of 11–15 points indicates poor sleep quality, and a total score ≥ 16 points indicates very poor sleep quality (29). The KAP sections employ five-point Likert scales, encompassing 10 questions in the knowledge dimension (with scores ranging from 10 to 50), 9 questions in the attitude dimension (with scores ranging from 9 to 45), and 6 questions in the practice dimension (with scores ranging from 6 to 30). Participants scoring above 80% of the total were designated as possessing sufficient knowledge, positive attitudes, and engaged in proactive practices. Individuals falling within the range of 60 to 80% of the total were classified as exhibiting moderate levels of knowledge, attitudes, and practices. Scores below 60% of the total indicated inadequate knowledge, negative attitudes, and inactive practices (30).

The questionnaires were uploaded onto *“Wen Juan Xing,”* an online platform dedicated to collecting survey data. With precision and care, they were distributed to the study participants through the widely employed WeChat platform.

### Sample size calculation

The sample size was calculated using the following formula:

$n={\left(Z_{\left(1-\alpha /2\right)}/\delta \right)}^{2}\times p\times \left(1-p\right)\text{,}$

where *n* represents the sample size, and *p* was assumed to be 0.5 to maximize the sample size. The significance level (α), or type I error, was set at 0.05, making Z_(1-α/2) = 1.96. The standard error (δ) was assumed to be 0.05. The final target was to collect at least 384 completed questionnaires.

### Statistical analysis

SPSS 26.0 (IBM, Armonk, NY, USA) and AMOS (IBM, Armonk, New York, USA) were used for statistical analysis. The continuous variables are presented as mean ± standard deviation (SD), and compared by Mann–Whitney test or Kruskal-Wallis H test. Categorical variables are presented as *n* (%). Spearman analysis was employed to evaluate the correlation among knowledge, attitude, and practice scores. And the univariate and multivariate logistics regression analysis was used to explore the factors associated with sleep quality. The Wald test was used to examine the regression coefficient. In the regression analysis, sleep quality was set as the dependent variable, while demographic characteristics and KAP scores were set as independent variables. To conduct binary logistic regression, sleep quality was converted into binary categorical variables. Individuals with a total PSQI score ≤ 10 were considered to have normal (include average or good) sleep quality, while those with a PSQI score > 10 were considered to have poor (include poor or very poor) sleep quality. Variables that demonstrated *p* < 0.05 in the univariate analysis were subsequently included in the multivariate regression model. A two-sided *p* < 0.05 was considered statistically significant.

## Results

A total of 441 questionnaires were collected for this study, with 18 instances of logical errors, resulting in 423 valid responses. Among these, 215 (50.83%) were aged 35 years or below, 280 (66.19%) were female, and 284 (67.14%) were physicians. Additionally, 386 (91.25%) reported no history of cardiovascular disease, 191 (45.15%) worked 1–2 night shifts weekly, 270 (63.83%) experienced chest tightness and palpitations during night shifts or insufficient sleep, and 128 (30.26%) suffered from chronic insomnia (Table 1).

**Table 1:** Demographic characteristics and KAP scores.

Characteristics	*N* (%)	Knowledge		Attitude		Practice	
		Score	*P*	Score	*P*	Score	*P*
Total score		43.23 ± 5.667		24.53 ± 4.591		20.22 ± 4.455	
Age, years			0.422		0.264		0.005
≤35	215 (50.83)	42.93 ± 6.22		24.24 ± 4.28		19.65 ± 4.55	
>35	208 (49.17)	43.54 ± 5.03		24.84 ± 4.88		20.82 ± 4.29	
Gender			0.138		0.003		0.420
Male	143 (33.81)	43.71 ± 5.681		23.79 ± 4.561		20.59 ± 4.812	
Female	280 (66.19)	42.99 ± 5.654		24.91 ± 4.567		20.04 ± 4.257	
Occupation			0.003		0.129		0.961
Doctor	284 (67.14)	43.63 ± 5.885		24.26 ± 4.457		20.20 ± 4.585	
Nurse	96 (22.70)	42.79 ± 5.287		24.98 ± 4.757		20.23 ± 3.954	
Other	43 (10.17)	41.56 ± 4.651		25.37 ± 5.000		20.35 ± 4.725	
Professional title			0.460		0.050		0.144
No Title	57 (13.48)	43.33 ± 6.128		25.37 ± 4.287		19.30 ± 5.398	
Junior	81 (19.15)	42.81 ± 5.523		24.02 ± 4.321		20.42 ± 4.404	
Intermediate	130 (30.73)	42.84 ± 6.243		23.88 ± 4.236		19.98 ± 4.206	
Senior	155 (36.64)	43.74 ± 5.04		25.04 ± 5.030		20.66 ± 4.280	
Residence			0.054		0.502		0.800
Non-urban	61 (14.42)	42.34 ± 5.026		24.2 ± 4.222		20.26 ± 4.355	
Urban	362 (85.58)	43.38 ± 5.761		24.59 ± 4.653		20.22 ± 4.477	
Hospital type			0.343		0.238		0.497
Tertiary	315 (74.47)	43.43 ± 5.759		24.61 ± 4.673		20.15 ± 4.617	
Secondary	73 (17.26)	42.89 ± 5.643		23.64 ± 3.776		20.29 ± 4.215	
Primary	14 (3.31)	42.43 ± 4.653		26.57 ± 5.893		21.43 ± 2.377	
Private and other	21 (4.96)	41.9 ± 4.969		25.19 ± 4.643		20.33 ± 3.903	
Education			0.021		0.216		0.124
College and below	34 (8.04)	41.26 ± 4.488		25.15 ± 4.001		19.97 ± 4.145	
Bachelor	194 (45.86)	43.4 ± 5.802		24.28 ± 4.939		20.73 ± 4.423	
Master and above	195 (46.10)	43.41 ± 5.675		24.68 ± 4.325		19.76 ± 4.505	
Cardiovascular disease			0.351		0.657		0.863
Yes	37 (8.75)	43.73 ± 6.239		24.41 ± 5.188		20.22 ± 4.732	
No	386 (91.25)	43.18 ± 5.615		24.55 ± 4.537		20.23 ± 4.433	
Average weekly night shift frequency			0.124		<0.001		0.690
<1 time	208 (49.17)	43.19 ± 5.23		25.63 ± 5.114		20.19 ± 4.371	
1 ~ 2 times	191 (45.15)	43.45 ± 6.158		23.45 ± 3.768		20.37 ± 4.627	
≥3 times	24 (5.67)	41.92 ± 5.25		23.67 ± 3.547		19.38 ± 3.786	
Experienced chest tightness, palpitations during night shifts or insufficient sleep			0.126		<0.001		0.007
Yes	270 (63.83)	43.46 ± 5.778		23.76 ± 4.317		19.77 ± 4.326	
No	153 (36.17)	42.82 ± 5.461		25.9 ± 4.751		21.02 ± 4.58	
Can you ensure 7–9 hours of sleep every day when not on night shifts?			0.339		<0.001		<0.001
Always (9–10 times every 10 days)	50 (11.82)	44.26 ± 4.379		26.62 ± 5.484		22.88 ± 4.676	
Often (7–8 times every 10 days)	89 (21.04)	43.19 ± 5.013		27.01 ± 4.916		20.93 ± 4.392	
Generally (4–6 times every 10 days)	124 (29.31)	42.6 ± 5.648		24.36 ± 3.523		20.32 ± 4.009	
Occasionally (1–3 times every 10 days)	123 (29.08)	43.56 ± 5.559		23.16 ± 3.729		18.94 ± 4.247	
Never	37 (8.75)	42.97 ± 8.477		20.89 ± 4.012		18.86 ± 4.590	
Frequency of sleep disturbance by external factors when not on night shift:			0.143		<0.001		0.225
Always (9–10 times every 10 days)	15 (3.55)	46 ± 3.742		21.27 ± 3.77		21.20 ± 5.130	
Often (7–8 times every 10 days)	73 (17.26)	42.01 ± 7.052		22.38 ± 3.71		19.16 ± 4.041	
Generally (4–6 times every 10 days)	103 (24.35)	43.54 ± 5.639		23.27 ± 3.603		20.50 ± 4.765	
Occasionally (1–3 times every 10 days)	192 (45.39)	43.52 ± 4.626		25.75 ± 4.708		20.47 ± 4.145	
Never	40 (9.46)	42.23 ± 7.43		27.10 ± 4.898		19.90 ± 5.339	
Frequency of sleep interruption due to personal reasons (such as palpitations, shortness of breath) when not on night shift:			0.478		<0.001		0.319
Always (9–10 times every 10 days)	6 (1.42)	38.83 ± 14.552		20.00 ± 1.549		24.83 ± 5.742	
Often (7–8 times every 10 days)	22 (5.20)	43.5 ± 5.918		21.23 ± 3.323		20.77 ± 5.740	
Generally (4–6 times every 10 days)	33 (7.80)	41.27 ± 7.129		22.91 ± 3.956		19.36 ± 3.757	
Occasionally (1–3 times every 10 days)	179 (42.32)	43.9 ± 4.536		23.57 ± 4.047		20.41 ± 4.206	
Never	183 (43.26)	43.04 ± 5.807		26.32 ± 4.708		19.98 ± 4.533	
Whether suffering from chronic insomnia:			0.216		<0.001		0.012
Yes	128 (30.26)	42.69 ± 5.812		22.02 ± 3.633		19.32 ± 3.882	
No	295 (69.74)	43.47 ± 5.596		25.63 ± 4.537		20.62 ± 4.633	

The mean knowledge, attitude, and practice scores were 43.23 ± 5.67 (possible range: 10–50), 24.53 ± 4.59 (possible 9–45), and 20.22 ± 4.455 (possible range: 6–30), respectively. The results showed that doctors (*p* = 0.003) and individuals with higher education (*p* = 0.021) were more likely to exhibit higher knowledge scores. Additionally, females (*p* = 0.003), those with fewer than one night shift per week (*p* < 0.001), absence of symptoms like chest tightness or palpitations (*p* < 0.001), regular 7–9 h of daily sleep (*p* < 0.001), undisturbed sleep (*p* < 0.001), and the absence of chronic insomnia (*p* < 0.001) were associated with higher attitude scores. Furthermore, participants over 35 years (*p* = 0.005), those without symptoms like chest tightness or palpitations (*p* = 0.007), consistent 7–9 h of daily sleep (*p* < 0.001), and without chronic insomnia (*p* = 0.012) were more likely to exhibit higher practice scores (Table 1).

Responses to the knowledge dimension showed that for *“I have systematically acquired professional knowledge related to sleep and cardiovascular health.”* (K1) and *“Excessive sleep duration may also increase the incidence and mortality risks of cardiovascular diseases.”* (K5), 46.58 and 35.23% got relatively low scores (chose “neutral” or “disagreed” or “strongly disagreed”), respectively (Supplementary Table S1). The attitude of healthcare workers revealed that, 31.68% were neutral, and 39.48% expressed negativity about whether they themselves receive adequate sleep (A1). Respectively, 52.71 and 68.08% reported that their sleep was influenced by related diseases (A4) and lifestyle habits (A5). Furthermore, 64.54 and 77.07% indicated that their sleep was affected by frequent night shifts (A6) and work-related stress (A7). These responses suggest that participants face challenges in enhancing their sleep due to life or work-related factors (Supplementary Table S2). In terms of practice, 13.48% of participants reported the necessity of pre-adjusting their routine to facilitate adaptation to night shifts (P1), 18.2% required several days to readjust to adequate sleep after night shifts (P2), and 16.78% resorted to medication to aid their sleep, a trend observed in a substantial portion of the population. Of equal concerning is the fact that 41.61% never undergo regular medical check-ups (P4), and 33.33% occasionally engage in healthy dietary practices and make efforts to improve unhealthy lifestyles (P7) (Supplementary Table S3).

Correlation analyses unveiled a negative correlation between knowledge and attitude (*r* = −0.116, *p* = 0.001), coupled with a positive correlation between knowledge and practice (*r* = 0.224, *p* < 0.001). Attitude displayed a negative correlation with sleep health awareness & habits (*r* = −0.158, *p* = 0.001) and PSQI (*r* = −0.388, *p* < 0.001). Furthermore, practice exhibited a negative correlation with PSQI (*r* = −0.252, *p* < 0.001), while sleep health awareness & habits were negatively correlated with PSQI (*r* = 0.423, *p* < 0.001) (Table 2).

**Table 2:** Correlation analysis.

	Knowledge	Attitude	Practice	Sleep health awareness and habits	PSQI
Knowledge	-				
Attitude	−0.116 (*p* = 0.017)	-			
Practice	0.224 (*p* < 0.001)	0.019 (*p* = 0.692)	-		
Sleep health awareness and habits	−0.080 (*p* = 0.099)	−0.158 (*p* = 0.001)	−0.084 (*p* = 0.085)	-	
Pittsburgh sleep quality index (PSQI)	−0.079 (*p* = 0.105)	−0.388 (*p* < 0.001)	−0.252 (*p* < 0.001)	0.423 (*p* < 0.001)	-

The multivariate analyses revealing that knowledge and practice scores did not exhibit independent associations with sleep quality. Simultaneously, attitude score (OR = 0.890, 95% CI: [0.807–0.981], *p* = 0.019), sleep health awareness & habits score (OR = 1.847, 95% CI: [1.284–2.656], *p* = 0.001), the experience of chest tightness or palpitations (OR = 6.084, 95% CI: [2.172–17.042], *p* = 0.001), and the presence of chronic insomnia (OR = 5.017, 95% CI: [2.428–10.368], *p* < 0.001) were independently associated with sleep quality (Table 3).

**Table 3:** Factors associated with sleep quality.

	Univariate		Multivariate	
	95%CI	*P*	95%CI	*P*
Knowledge score	0.979 (0.936–1.024)	0.353		
Attitude score	0.792 (0.728–0.862)	<0.001	0.890 (0.807–0.981)	0.019
Practice score	0.909 (0.852–0.970)	0.004	0.925 (0.853–1.003)	0.059
Sleep health awareness and habits score	2.213 (1.643–2.982)	<0.001	1.847 (1.284–2.656)	0.001
Age, years				
≤35	REF			
>35	0.828 (0.482–1.423)	0.494		
Gender				
Male	REF			
Female	1.419 (0.780–2.582)	0.252		
Occupation type				
Doctor	REF			
Nurse	1.605 (0.885–2.911)	0.119		
Other	0.298 (0.069–1.279)	0.103		
Professional title				
No title	REF			
Junior	2.762 (0.955–7.991)	0.061		
Intermediate	1.780 (0.630–5.030)	0.277		
Associate senior and above	1.630 (0.584–4.549)	0.351		
Residence				
Non-urban	REF			
Urban	1.162 (0.523–2.579)	0.713		
Hospital type				
Tertiary	REF			
Other	1.232 (0.677–2.240)	0.494		
Education level				
College and below	REF			
Bachelor’s	1.189 (0.429–3.298)	0.740		
Master’s and above	0.814 (0.287–2.305)	0.698		
Cardiovascular disease				
Yes	2.379 (1.088–5.201)	0.030	2.248 (0.830–6.089)	0.111
No	REF		REF	
Average weekly night shift frequency				
<1 time	REF			
≥1 time	1.408 (0.816–2.428)	0.219		
Experienced chest tightness, palpitations, during night shifts or insufficient sleep				
Yes	7.921 (3.101–20.236)	<0.001	6.084 (2.172–17.042)	0.001
No	REF		REF	
Can you ensure 7–9 hours of sleep every day when not on night shifts?				
No	REF		REF	
Yes	0.310 (0.146–0.656)	0.002	0.977 (0.379–2.520)	0.961
Frequency of sleep disturbance by external factors when not on night shift				
No	REF			
Yes	3.529 (0.829–15.022)	0.088		
Frequency of sleep interruption due to personal reasons (such as palpitations, shortness of breath) when not on night shift				
No	REF		REF	
Yes	2.727 (1.472–5.055)	0.001	0.863 (0.395–1.885)	0.711
Whether suffering from chronic insomnia				
Yes	10.831 (5.759–20.370)	<0.001	5.017 (2.428–10.368)	<0.001
No	REF		REF	

## Discussion

This study reveals that healthcare workers exhibit satisfactory knowledge, negative attitudes, and moderate practices concerning sleep hygiene and cardiovascular health. In light of these findings, we recommend the implementation of tailored interventions and educational programs to improve the attitudes and practices of healthcare workers. Ultimately, this approach aims to improve sleep hygiene and foster better cardiovascular health outcomes within this population.

The findings of this study shed light on the complex landscape of healthcare workers’ perspectives and practices regarding sleep hygiene and cardiovascular health. These findings align with prior research that emphasizes the importance of understanding healthcare professionals’ awareness and practices in promoting overall well-being. Sleep-related issues, such as mental fatigue, can adversely affect the physical health of healthcare workers, potentially leading to increased job burnout and impacting personal career achievements (31). In examining demographic and occupational factors, age emerged as a significant determinant, with individuals aged 35 and below exhibiting higher mean practice scores. This contrasts with the conclusions drawn in previous studies, which suggest that a higher proportion of young people have unhealthy lifestyle habits (32). Furthermore, the notable gender differences, where female healthcare workers demonstrated more positive attitudes, validate existing research emphasizing gender-specific approaches to healthcare education (33). Occupational distinctions were evident, with doctors showing higher knowledge scores and nurses displaying more positive attitudes.

Correlation analyses provided valuable insights into the interconnectedness of knowledge, attitudes, practices, and sleep quality. The negative correlation between knowledge and attitude is consistent with studies highlighting the intricate balance between knowledge acquisition and the formation of attitudes (34). The positive correlation between knowledge and practice underscores the potential impact of informed decision-making on translating knowledge into practical habits. The negative correlation of attitude with sleep health awareness & habits and PSQI, as well as the positive correlation of practice with PSQI and negative correlation with sleep health awareness & habits, elucidate the complex interplay between attitudes, practices, and sleep outcomes. The negative correlation between knowledge and attitude in our study could stem from psychological responses like stress or guilt from increased sleep hygiene awareness, cognitive dissonance from misaligned behaviors and knowledge, cultural influences on sleep attitudes, and personal beliefs prioritizing activities over sleep despite health knowledge. Multivariate analyses, delving into factors independently associated with sleep quality, disclosed intriguing patterns. It is noteworthy that the study reveals knowledge and practice do not significantly impact sleep quality. Factors such as work-related stress, irregular work schedules, or specific challenges within the healthcare industry may play a more crucial role in shaping the sleep patterns of healthcare professionals. Besides, attitude score, sleep health awareness & habits score, the experience of chest tightness or palpitations during night shifts, and the presence of chronic insomnia were identified as independent predictors of sleep quality. This refined understanding aligns with studies that emphasize the subjective and psychological dimensions of sleep quality (35). The identified independent association of attitudes, sleep health awareness & habits, and specific health conditions with sleep quality underscores the necessity for focused interventions that address the holistic well-being of healthcare workers.

The survey results indicate notable patterns in healthcare workers’ knowledge, attitudes, and practices regarding sleep and cardiovascular health. In terms of knowledge, a substantial portion of participants acknowledged the relationship between sleep and cardiovascular diseases, recognizing factors such as insufficient sleep duration, irregular sleep patterns, and lifestyle habits as contributors to increased cardiovascular risks. However, there appears to be less awareness regarding the risks associated with excessive sleep duration. Educational initiatives should focus on addressing specific gaps in knowledge, such as the potential risks associated with excessive sleep duration and the intricate mechanisms through which insufficient sleep impacts cardiovascular health (36, 37).

In the evaluation of attitudes, a majority of participants conveyed a robust belief in the significance of adequate and consistent sleep for maintaining cardiovascular health. Nevertheless, a significant number reported challenges related to work intensity and stress, influencing their sleep patterns. This resonates with studies highlighting the impact of work-related stress on sleep quality and the challenges healthcare workers face in maintaining healthy sleep habits (38, 39). Additionally, the reliance on sleep aids among healthcare workers raises concerns, as it may indicate underlying issues that require targeted interventions. Given the reported challenges related to work intensity and stress, interventions should incorporate stress management techniques and promote a healthy work-life balance. Furthermore, the use of sleep aids among healthcare workers necessitates careful examination, and interventions should prioritize non-pharmacological approaches, including cognitive-behavioral therapy for insomnia, which has shown efficacy in improving sleep quality without relying on medications (40).

Regarding practices, participants exhibited variability in their adherence to sleep-promoting behaviors. While many engaged in self-monitoring of cardiovascular indicators and underwent regular health check-ups, a significant number faced challenges in adjusting their schedules before and after night shifts. This aligns with existing literature that underscores the challenges healthcare workers confront in adapting their sleep patterns to irregular work schedules, thereby impairing their overall sleep quality and health (41, 42). To address the observed difficulties in adjusting schedules before and after night shifts, organizational policies should be revised to optimize shift scheduling and mitigate the negative impact on sleep quality. Interventions should also encourage the adoption of evidence-based sleep-promoting practices, such as strategic napping, which has been shown to improve alertness and performance among shift workers (43). Additionally, fostering a culture of comprehensive well-being within healthcare settings may involve creating designated areas for rest and relaxation, providing resources for stress reduction, and promoting teamwork to manage workload effectively.

### Limitations and future perspectives

While this study provides valuable insights, it is essential to acknowledge certain limitations. The cross-sectional design restricts the ability to establish causal relationships, as it only permits the observation of associations at a specific point in time, without determining the direction of causality. Additionally, the reliance on self-reported data introduces the potential for response bias, which may affect the accuracy of the findings. The single-center nature of the study, specifically limited to health workers from Yancheng TCM Hospital affiliated with Nanjing University of Chinese Medicine, further constrains the generalizability of the results to other healthcare settings or broader populations. Moreover, the lack of control for potential confounding variables, such as work stress, workload, or socioeconomic factors, could influence the observed associations, highlighting the need for future research to address these factors to enhance the validity of the findings. Employing longitudinal designs and objective measures in future studies could provide a more nuanced understanding of these associations. Despite these limitations, the study’s strengths—including a substantial sample from a reputable healthcare institution, a comprehensive assessment methodology, and robust statistical analyses—underscore the credibility of our findings. These results significantly contribute to the understanding of healthcare workers’ perceptions, sleep hygiene, and cardiovascular health, providing a foundation for targeted interventions and guiding future research efforts in this critical domain.

### Practical implications

This study’s findings carry important implications for healthcare practice and the well-being of healthcare workers. The significant associations between negative attitudes, work-related stress, chest tightness or palpitations, and chronic insomnia suggest that healthcare institutions must implement structured interventions to mitigate these effects. Tailored training programs focusing on improving attitudes toward sleep hygiene and cardiovascular health should be integrated into healthcare professional development. Additionally, management strategies that include regular health assessments, stress reduction techniques, and support for night-shift workers can help in addressing sleep disturbances. Importantly, providing healthcare professionals with non-pharmacological interventions like cognitive behavior therapy for insomnia can promote healthier sleep patterns without reliance on medication. Institutions should also revise policies to create more favorable work conditions, including improving shift scheduling to reduce the negative impact on sleep. By addressing these practical aspects, healthcare workers’ well-being can be safeguarded, potentially leading to better patient care outcomes.

## Conclusion

In conclusion, healthcare workers exhibited satisfactory knowledge, unfavorable attitudes, and moderate practices concerning sleep hygiene and cardiovascular health. Recognizing the identified negative attitudes among healthcare workers, it is recommended that targeted interventions and educational programs be implemented to improve their attitudes and practices regarding sleep hygiene. These interventions should be tailored to address the specific negative attitudes and practices identified, ensuring they are directly relevant and applicable to the healthcare settings. This may involve integrating sleep hygiene education into professional development courses and wellness programs for healthcare workers, ultimately enhancing their overall well-being and potentially contributing to improved patient care. As traditional Chinese medicine says, “Qi Ju You Chang,” which means “Regular living habits.”

## Data Availability

The original contributions presented in the study are included in the article/Supplementary material, further inquiries can be directed to the corresponding author.
