# Supplementary material for: Knowledge, attitude, and practice toward sleep hygiene and cardiovascular health: a cross-sectional survey among healthcare workers
Source: Front Public Health. 2024 Oct 17;12:1415849. doi: 10.3389/fpubh.2024.1415849 (PMC11524854; doi:10.3389/fpubh.2024.1415849)
Supplement: Supplementary file 1 [file Table_1.docx]

**Table S1. Knowledge Section Responses**

| **Knowledge** | **N (%)** | | | | |
| --- | --- | --- | --- | --- | --- |
|  | **Strongly Agree** | **Agree** | **Neutral** | **Disagree** | **Strongly Disagree** |
| **1.** **I have systematically acquired professional knowledge related to sleep and cardiovascular health.** | 88(20.8) | 138(32.62) | 121(28.61) | 57(13.48) | 19(4.49) |
| **2.** **Sleep disorders (such as insomnia) and poor sleep habits (duration and regularity) can increase the risk of CVD.** | 239(56.5) | 160(37.83) | 14(3.31) | 8(1.89) | 2(0.47) |
| **3.** **Insufficient sleep duration may increase the risk of diseases such as coronary heart disease, hypertension, arrhythmias, and heart failure.** | 248(58.63) | 155(36.64) | 13(3.07) | 5(1.18) | 2(0.47) |
| **4.** **For patients already diagnosed with cardiovascular diseases, insufficient sleep duration may increase the risk of onset/death.** | 244(57.68) | 154(36.41) | 18(4.26) | 6(1.42) | 1(0.24) |
| **5.** **Excessive sleep duration may also increase the incidence and mortality risks of cardiovascular diseases.** | 123(29.08) | 151(35.7) | 104(24.59) | 37(8.75) | 8(1.89) |
| **6.** **Insufficient sleep may increase the risk of CVD by triggering metabolic disorders.** | 224(52.96) | 180(42.55) | 12(2.84) | 4(0.95) | 3(0.71) |
| **7.** **Insufficient sleep may accelerate blood vessel aging and increase the risk of CVD by affecting endothelial function.** | 230(54.37) | 175(41.37) | 12(2.84) | 3(0.71) | 3(0.71) |
| **8.** **Insufficient sleep may increase the risk of CVD by impacting neuroimmune function and accelerating atherosclerosis.** | 226(53.43) | 176(41.61) | 14(3.31) | 3(0.71) | 4(0.95) |
| **9.** **Irregular sleep patterns (such as staying up late or working night shifts) may disrupt circadian rhythms, thereby increasing cardiovascular risks.** | 249(58.87) | 159(37.59) | 8(1.89) | 4(0.95) | 3(0.71) |
| **10.** **Improving sleep duration and regularity can enhance the prognosis of patients with CVD.** | 244(57.68) | 152(35.93) | 17(4.02) | 4(0.95) | 6(1.42) |
