# Supplementary material for: Knowledge, attitude, and practice toward sleep hygiene and cardiovascular health: a cross-sectional survey among healthcare workers
Source: Front Public Health. 2024 Oct 17;12:1415849. doi: 10.3389/fpubh.2024.1415849 (PMC11524854; doi:10.3389/fpubh.2024.1415849)
Supplement: Supplementary file 2 [file Table_2.docx]

**Table S2. Attitude Section Responses**

| **Attitude** | **Strongly Agree** | **Agree** | **Neutral** | **Disagree** | **Strongly Disagree** |
| --- | --- | --- | --- | --- | --- |
| **1.I obtain sufficient and regular sleep.** | 41(9.69) | 81(19.15) | 134(31.68) | 122(28.84) | 45(10.64) |
| **2.Adequate sleep is crucial for maintaining cardiovascular health.** | 263(62.17) | 147(34.75) | 8(1.89) | 5(1.18) | (0) |
| **3.During rest, I prioritize ensuring sufficient time for sleep.** | 123(29.08) | 168(39.72) | 110(26) | 20(4.73) | 2(0.47) |
| **4.My sleep is influenced by disorders such as insomnia and sleep-wake rhythm disturbances.** | 88(20.8) | 135(31.91) | 85(20.09) | 81(19.15) | 34(8.04) |
| **5.My sleep is influenced by lifestyle habits (such as staying up late or using electronic devices).** | 102(24.11) | 186(43.97) | 95(22.46) | 33(7.8) | 7(1.65) |
| **6.Frequent night shifts prevent me from obtaining sufficient and regular sleep.** | 143(33.81) | 130(30.73) | 94(22.22) | 38(8.98) | 18(4.26) |
| **7.Work-related stress affects my sleep.** | 134(31.68) | 192(45.39) | 65(15.37) | 27(6.38) | 5(1.18) |
| **8.Given the current intensity of my work, it is challenging to ensure sufficient and regular sleep.** | 123(29.08) | 155(36.64) | 94(22.22) | 41(9.69) | 10(2.36) |
| **9.Given the current intensity of my work, ensuring sufficient and regular sleep inevitably requires sacrificing personal time (such as socializing, entertainment, etc.).** | 147(34.75) | 187(44.21) | 54(12.77) | 31(7.33) | 4(0.95) |
| **10.Using sleep aids helps ensure sufficient and regular sleep.** | 74(17.49) | 158(37.35) | 114(26.95) | 61(14.42) | 16(3.78) |
