# Supplementary material for: Knowledge, attitude, and practice toward sleep hygiene and cardiovascular health: a cross-sectional survey among healthcare workers
Source: Front Public Health. 2024 Oct 17;12:1415849. doi: 10.3389/fpubh.2024.1415849 (PMC11524854; doi:10.3389/fpubh.2024.1415849)
Supplement: Supplementary file 3 [file Table_3.docx]

**Table S3. Practice Section Responses**

| **Practice** | **Always** | **Often** | **Neutral** | **Occasionally** | **Never** |
| --- | --- | --- | --- | --- | --- |
| **1.Before night shifts, do you adjust your schedule in advance to help your body adapt?** | 57(13.48) | 87(20.57) | 106(25.06) | 130(30.73) | 43(10.17) |
| **2.After night shifts, do you adjust your schedule in the following days to ensure sufficient sleep?** | 77(18.2) | 154(36.41) | 107(25.3) | 57(13.48) | 28(6.62) |
| **3.When having difficulty falling asleep, do you use medication to assist with sleep?** | 71(16.78) | 149(35.22) | 116(27.42) | 61(14.42) | 26(6.15) |
| **4.Do you undergo regular health check-ups?** | 34(8.04) | 79(18.68) | 53(12.53) | 81(19.15) | 176(41.61) |
| **5.Do you self-monitor indicators related to cardiovascular diseases, such as weight, blood pressure, and blood sugar?** | 102(24.11) | 136(32.15) | 112(26.48) | 46(10.87) | 27(6.38) |
| **6.Do you engage in regular aerobic exercise?** | 86(20.33) | 138(32.62) | 118(27.9) | 57(13.48) | 24(5.67) |
| **7.Do you maintain a healthy diet and work on improving unhealthy lifestyle habits?** | 41(9.69) | 67(15.84) | 148(34.99) | 141(33.33) | 26(6.15) |
