# Supplementary material for: Knowledge, attitude, and practice toward sleep hygiene and cardiovascular health: a cross-sectional survey among healthcare workers
Source: Front Public Health. 2024 Oct 17;12:1415849. doi: 10.3389/fpubh.2024.1415849 (PMC11524854; doi:10.3389/fpubh.2024.1415849)
Supplement: Supplementary file 4 [file Table_4.docx]

| Dear Participants,  We are researchers from * Hospital, sincerely inviting you to participate in our research project. The aim of this study is to understand the knowledge, attitudes, and practices of healthcare professionals regarding sleep deprivation and cardiovascular diseases. The insights gained will serve as a foundation for developing scientifically based intervention strategies, potentially benefiting more individuals in the future and improving their health conditions. Your participation in this study is voluntary, and if you agree to participate, please refer to the following instructions.  1. Please complete the questionnaire. There are no right or wrong answers; you only need to provide responses based on your actual circumstances. If you have any questions during the answering process, feel free to reach out to us. Once completed, please submit it promptly.  2. This study is a simple questionnaire survey that will not cause harm to your physical or mental well-being. However, it will involve some privacy-related questions, such as your gender and age. We assure you that your information will be kept strictly confidential and will not be disclosed. Please feel free to fill in the questionnaire with confidence.  3.As a participant, you can inquire about information related to this study and its progress at any time. If you decide to withdraw from the study, please inform us, and your data will not be included in the research results.  Finally, we sincerely appreciate your time and support for our scientific research amidst your busy schedule!  □ I am aware of and agree to have the collected data used for scientific research.  Informed Consent Signature: |
| --- |

| **Part I-Basic Information** | |
| --- | --- |
| **1.** **Your age:** | years |
| **2.** **Your gender:** | a. Male  b. Female |
| **3.** **Your occupation type:** | a. Doctor  b. Nurse |
| **4.** **Your professional title:** | a. No title  b. Junior  c. Intermediate  d. Associate Senior  e. Senior |
| **5.** **Your residence:** | a. Rural  b. Urban  c. Suburban |
| **6.** **The type of hospital you work at:** | a. Tertiary hospital  b. Secondary hospital  c. Primary hospital  d. Private hospital  e. Other |
| **7. Your educational level:** | a. College and below  b. Bachelor  c. Master's and above |
| **8.** **Do you have cardiovascular disease?** | a.Yes  b.No |
| **9.** **Average frequency of night shifts per week:** | a. <1 time (Not every week and less than once a week on average)  b.1~2 times  c.≥3 times |
| **10.** **Have you experienced symptoms such as chest tightness or palpitations during night shifts or insufficient sleep?** | a. Yes  b. No |
| **11.** **When not on night shifts, can you ensure 7-9 hours of sleep per day?** | a. Always (9-10 times every 10 days)  b. Often (7-8 times every 10 days)  c. Generally (4-6 times every 10 days)  d. Occasionally (1-3 times every 10 days)  E. Never |
| **12.** **When not on night shifts, how often is your sleep disturbed by external factors (such as work or family matters)?** | a. Always (9-10 times every 10 days)  b. Often (7-8 times every 10 days)  c. Generally (4-6 times every 10 days)  d. Occasionally (1-3 times every 10 days)  E. Never |
| **13.** **When not on night shifts, how often is your sleep interrupted due to personal reasons (such as palpitations or shortness of breath)?** | a. Always (9-10 times every 10 days)  b. Often (7-8 times every 10 days)  c. Generally (4-6 times every 10 days)  d. Occasionally (1-3 times every 10 days)  E. Never |
| **14.** **Do you suffer from chronic insomnia?** | a.Yes  b.No |
| **15.15*6=80** | a.True  b.False  c.Uncertain |

**Part II-Knowledge**

**Please select one option from "Strongly Agree" to "Strongly Disagree" based on your agreement with the statements.**

| **1. I have systematically acquired professional knowledge related to sleep and cardiovascular health.** | **a. Strongly Agree** | **B. Agree** | **C. Neutral** | **b. Disagree** | **c. Strongly Disagree** |
| --- | --- | --- | --- | --- | --- |
| **2. Sleep disorders (such as insomnia) and poor sleep habits (duration and regularity) can increase the risk of CVD.** | **a. Strongly Agree** | **B. Agree** | **C. Neutral** | **b. Disagree** | **c. Strongly Disagree** |
| **3. Insufficient sleep duration may increase the risk of diseases such as coronary heart disease, hypertension, arrhythmias, and heart failure.** | **a. Strongly Agree** | **B. Agree** | **C. Neutral** | **b. Disagree** | **c. Strongly Disagree** |
| **4. For patients already diagnosed with cardiovascular diseases, insufficient sleep duration may increase the risk of onset/death.** | **a. Strongly Agree** | **B. Agree** | **C. Neutral** | **b. Disagree** | **c. Strongly Disagree** |
| **5. Excessive sleep duration may also increase the incidence and mortality risks of cardiovascular diseases.** | **a. Strongly Agree** | **B. Agree** | **C. Neutral** | **b. Disagree** | **c. Strongly Disagree** |
| **6. Insufficient sleep may increase the risk of CVD by triggering metabolic disorders.** | **a. Strongly Agree** | **B. Agree** | **C. Neutral** | **b. Disagree** | **c. Strongly Disagree** |
| **7. Insufficient sleep may accelerate blood vessel aging and increase the risk of CVD by affecting endothelial function.** | **a. Strongly Agree** | **B. Agree** | **C. Neutral** | **b. Disagree** | **c. Strongly Disagree** |
| **8. Insufficient sleep may increase the risk of CVD by impacting neuroimmune function and accelerating atherosclerosis.** | **a. Strongly Agree** | **B. Agree** | **C. Neutral** | **b. Disagree** | **c. Strongly Disagree** |
| **9. Irregular sleep patterns (such as staying up late or working night shifts) may disrupt circadian rhythms, thereby increasing cardiovascular risks.** | **a. Strongly Agree** | **B. Agree** | **C. Neutral** | **b. Disagree** | **c. Strongly Disagree** |
| **10. Improving sleep duration and regularity can enhance the prognosis of patients with CVD.** | **a. Strongly Agree** | **B. Agree** | **C. Neutral** | **b. Disagree** | **c. Strongly Disagree** |

**Part-III Attitude**

**Please choose one option from "Strongly Agree" to "Strongly Disagree" based on your agreement with the statements.**

| **1.I obtain sufficient and regular sleep.** | **a. Strongly Agree** | **b. Agree** | **c. Neutral** | **d. Disagree** | **e. Strongly Disagree** |
| --- | --- | --- | --- | --- | --- |
| **2.My sleep is influenced by disorders such as insomnia and sleep-wake rhythm disturbances.** | **a. Strongly Agree** | **b. Agree** | **c. Neutral** | **d. Disagree** | **e. Strongly Disagree** |
| **3.My sleep is influenced by lifestyle habits (such as staying up late or using electronic devices).** | **a. Strongly Agree** | **b. Agree** | **c. Neutral** | **d. Disagree** | **e. Strongly Disagree** |
| **4.Frequent night shifts prevent me from obtaining sufficient and regular sleep.** | **a. Strongly Agree** | **b. Agree** | **c. Neutral** | **d. Disagree** | **e. Strongly Disagree** |
| **5.Work-related stress affects my sleep.** | **a. Strongly Agree** | **b. Agree** | **c. Neutral** | **d. Disagree** | **e. Strongly Disagree** |
| **6.Given the current intensity of my work, it is challenging to ensure sufficient and regular sleep.** | **a. Strongly Agree** | **b. Agree** | **c. Neutral** | **d. Disagree** | **e. Strongly Disagree** |
| **7.Given the current intensity of my work, ensuring sufficient and regular sleep inevitably requires sacrificing personal time (such as socializing, entertainment, etc.).** | **a. Strongly Agree** | **b. Agree** | **c. Neutral** | **d. Disagree** | **e. Strongly Disagree** |
| **8.Using sleep aids helps ensure sufficient and regular sleep.** | **a. Strongly Agree** | **b. Agree** | **c. Neutral** | **d. Disagree** | **e. Strongly Disagree** |
| **9.The cardiovascular disease risk caused by insufficient sleep can be alleviated through compensatory sleep.** | **a. Strongly Agree** | **b. Agree** | **c. Neutral** | **d. Disagree** | **e. Strongly Disagree** |

**Part IV- Practice**

**Please choose the option that best describes your behavior in the given situations.**

| **1.Before night shifts, do you adjust your schedule in advance to help your body adapt?** | **a. Always** | **b. Often** | **c. Sometimes** | **d. Occasionally** | **e. Never** |
| --- | --- | --- | --- | --- | --- |
| **2.After night shifts, do you adjust your schedule in the following days to ensure sufficient sleep?** | **a. Always** | **b. Often** | **c. Sometimes** | **d. Occasionally** | **e. Never** |
| **3.When having difficulty falling asleep, do you use medication to assist with sleep?** | **a. Always** | **b. Often** | **c. Sometimes** | **d. Occasionally** | **e. Never** |
| **4.Do you undergo regular health check-ups?** | **a. Always** | **b. Often** | **c. Sometimes** | **d. Occasionally** | **e. Never** |
| **5.Do you self-monitor indicators related to cardiovascular diseases, such as weight, blood pressure, and blood sugar?** | **a. Always** | **b. Often** | **c. Sometimes** | **d. Occasionally** | **e. Never** |
| **6.Do you engage in regular aerobic exercise?** | **a. Always** | **b. Often** | **c. Sometimes** | **d. Occasionally** | **e. Never** |
| **7.Do you maintain a healthy diet and work on improving unhealthy lifestyle habits?** | **a. Always** | **b. Often** | **c. Sometimes** | **d. Occasionally** | **e. Never** |

**Part V - Sleep Hygiene Awareness and Habits**

**Please provide a number based on the average number of days per week (0-7) you engage in or experience the following activities:**

| **1.** **Taking a nap or dozing off** |  |
| --- | --- |
| **2.** **Feeling thirsty when going to bed** |  |
| **3.** **Feeling hungry when going to bed** |  |
| **4.** **Smoking more than one pack of cigarettes per day** |  |
| **5.** **Regularly taking sleep-inducing medication** |  |
| **6.** **Consuming caffeinated beverages within 4 hours before bedtime** |  |
| **7.** **Consuming alcohol within 2 hours before bedtime** |  |
| **8.** **Taking medications containing caffeine within 4 hours before bedtime** |  |
| **9.** **Worrying about the ability to sleep before going to bed** |  |
| **10.** **Worrying about the ability to sleep at night during the day** |  |
| **11.** **Using alcohol to aid sleep** |  |
| **12.** **Engaging in vigorous physical activity within 2 hours before bedtime** |  |
| **13.** **Being disturbed by light while sleeping** |  |
| **14.** **Being disturbed by noise while sleeping** |  |
| **15.** **Being disturbed by bed partners while sleeping** |  |
| **16.** **Sleeping at the same time every night** |  |
| **17.** **Trying to relax before bedtime** |  |
| **18.** **Exercising in the afternoon or evening** |  |
| **19.** **Ensuring the bedroom or bed is warm and comfortable when sleeping** |  |

**Part VI-- Pittsburgh Sleep Quality Index (PSQI)**

**Please select or provide the most accurate answers based on your sleep patterns in the past month:**

| **What time do you usually go to bed at night in the past month? (24-hour clock, e.g., 23:30)** |  | | | |
| --- | --- | --- | --- | --- |
| **How long does it usually take you to fall asleep after getting into bed in the past month?** | **a. Within 15 minutes** | **b. 16-30 minutes** | **c. 31-60 minutes** | **d. 60 minutes or more** |
| **What time do you usually get up in the morning in the past month? (24-hour clock, e.g., 07:00)** |  | | | |
| **How many hours of actual sleep do you get at night on average in the past month? (Not equal to time spent in bed, e.g., 6.5)** |  | | | |
| **In the past month, how often have you been bothered by the following:** |  |  |  |  |
| **Difficulty falling asleep (within 30 minutes)** | **a. None** | **b. Less than 1 time per week** | **c. 1-2 times per week** | **d. 3 times or more per week** |
| **Waking up in the middle of the night or early morning** | **a. None** | **b. Less than 1 time per week** | **c. 1-2 times per week** | **d. 3 times or more per week** |
| **Needing to get up to use the bathroom** | **a. None** | **b. Less than 1 time per week** | **c. 1-2 times per week** | **d. 3 times or more per week** |
| **Breathing difficulties** | **a. None** | **b. Less than 1 time per week** | **c. 1-2 times per week** | **d. 3 times or more per week** |
| **Coughing or snoring loudly** | **a. None** | **b. Less than 1 time per week** | **c. 1-2 times per week** | **d. 3 times or more per week** |
| **Feeling cold** | **a. None** | **b. Less than 1 time per week** | **c. 1-2 times per week** | **d. 3 times or more per week** |
| **Feeling hot** | **a. None** | **b. Less than 1 time per week** | **c. 1-2 times per week** | **d. 3 times or more per week** |
| **Having bad dreams** | **a. None** | **b. Less than 1 time per week** | **c. 1-2 times per week** | **d. 3 times or more per week** |
| **Pain or discomfort** | **a. None** | **b. Less than 1 time per week** | **c. 1-2 times per week** | **d. 3 times or more per week** |
| **Other things that affected your sleep** | **a. None** | **b. Less than 1 time per week** | **c. 1-2 times per week** | **d. 3 times or more per week** |
| **Overall, how would you rate your sleep quality in the past month?** | **a.** **Very good** | **b.** **Fairly good** | **c. Fairly poor** | **d. Very poor** |
| **In the past month, how often have you taken sleeping medication?** | **a. None** | **b. Less than 1 time per week** | **c. 1-2 times per week** | **d. 3 times or more per week** |
| **In the past month, how often have you felt tired?** | **a. None** | **b. Less than 1 time per week** | **c. 1-2 times per week** | **d. 3 times or more per week** |
| **In the past month, how often have you had trouble staying awake while doing things?** | **a. Never** | **b. Occasionally** | **c. Sometimes** | **d. Often** |
